# Editorial: Progenitor diversity and neural cell specification in the central nervous system

**DOI:** 10.3389/fncel.2015.00358

**Published:** 2015-09-09

**Authors:** Caroline Rouaux, Cecília Hedin-Pereira, Marcos R. Costa

**Affiliations:** ^1^Institut National de la Santé et de la Recherche Médical U1118, Fédération de Médecine Translationnelle de Strasbourg, Université de StrasbourgStrasbourg, France; ^2^Institute of Biophysics Carlos Chagas Filho, Federal University of Rio de JaneiroRio de Janeiro, Brazil; ^3^Brain Institute, Federal University of Rio Grande do NorteNatal, Brazil

**Keywords:** central nervous system, cellular diversity, progenitor cells, neurons, glial cells, fate specification

The central nervous system (CNS) harbors an enormous diversity of neuronal and glial cell types, which can be identified according to morphological, chemical, and electrical properties. This variety of cell types is generated from progenitor cells located in different germinative niches, where distinct molecular signalings prompt distinctive transcription factors expression. In the last two decades, it has been acknowledged that such progenitor diversity is important for the generation of different subtypes of neurons, astrocytes and oligodendrocytes. Genetic fate-mapping studies have provided direct evidence for the contribution of separate cohorts of progenitor cells to generate individual subtypes of neurons and/or glial cells. Additionally, genetic deletions of single transcription factors and forced expression of ectopic transcription factor genes have pointed out the leading role of such molecules on the specification of different, individual cell types. However, other sources of data indicate that the environment plays important roles in cellular specification during CNS development, eventually overriding the influence of early transcription factors expression. These observations suggest that genetic determinants for both neuronal and glial specification would be changeable. However, the time-window during which neuronal and glial lineage genetic programs could be overwritten by external signals remains to be determined, along with exact signals that could perform such a fate modification. This research topic gathers a number of articles highlighting the role of a wide panel of intrinsic and extrinsic factors that contribute to the generation of such diversity. More importantly, the possible interplay between intrinsic and extrinsic factors is here considered in a new light, in which intrinsic and extrinsic signals act not only in concert, but also within a critical time-window, to determine cell fate.

Focusing on the specification of glutamatergic and GABAergic neurons in the developing cerebral cortex, Costa & Müller and Brandão & Romcy-Pereira discuss the evidences supporting the existence of genetically committed progenitor populations contributing to the generation of discrete neuronal subtypes (Costa and Muller, 2014; Brandão and Romcy-Pereira, 2015). In the first article, the authors discuss classical experiments of cell-lineage tracing in the developing cerebral cortex and try to conciliate data from these first studies with more recent genetic fate-mapping works that suggest the existence of multipotent and fate-restricted progenitors for glutamatergic cortical neurons. The second article reviews evidences for the generation of discrete GABAergic cortical neurons from separate progenitor territories. In addition, both articles discuss the important role of environmental signals and activity-dependent mechanisms in regulating the fate of cortical neurons, supporting the view that intrinsic genetic mechanisms are not the sole determinants of neuronal phenotypes. According to this notion, the article by Vasconcelos and Castro highlights a previously unexpected degree of complexity provided by the oscillatory expression of proneural genes in response to external signals (Vasconcelos and Castro, 2014).

This complex scenario of interactions is not restricted to developmental neurogenesis but extends to adulthood. Sequerra discusses the maintenance of morphogenetic gradients in the adult subventricular zone (SVZ), regulating the expression of genes involved in neuronal specification (Sequerra, 2014). He also points out the higher complexity of the adult neurogenic milieu as compared to the embryonic, which could widen the range of possible cellular phenotypes. In consonance with this article, Bernal and colleagues show mechanisms of extrinsic lipid signaling regulating the expression of transcription factors involved in the balance between neural progenitor cell proliferation and differentiation in the adult SVZ (Bernal et al., 2015).

Neuronal diversity may only be the tip of the iceberg, considering the emerging complexity of glial cells as revealed in the articles by Schitine et al. and Tomassy and Fossati (Tomassy and Fossati, 2014; Schitine et al., 2015). Astrocyte heterogeneity, regarding morphology and functions, is still a matter of debate in the literature. The first article reviews the diverse origins of these cells and the possibility that this may reflect in heterogenous astrocyte types, making the specific contributions of these cells to the brain functions even more complex (Schitine et al., 2015). The authors also review the morphological changes and peculiar responses of astrocytic subpopulations associated to different diseases, unveiling the relation between astrocytic diversity and environmental differences in pathological conditions. Supporting the notion that extrinsic signals may be pivotal in controlling astroglial progenitor behavior through the regulation of intrinsic genetic mechanisms, Stipursky et al., in their original paper, reveal the role TGF-beta in controlling glial progenitor differentiation (Stipursky et al., 2014). The authors show that TGF-beta down regulates the expression of FoxG1 and causes premature transformation of radial glial cells, which also leads to alterations in the laminar distribution of neurons. Likewise, Schmid et al. show that the disruption of the radial glia scaffold upon genetic deletion of alpha-catenin leads to a severe disruption of the laminar organization of cortical neurons, mimicking the human subcortical band heterotopia (Schmid et al., 2014).

The review by Tomassy and Fossati discusses the different origins of oligodendrocyte progenitors and puts forward the appealing question as to which extent this diverse origin could result in the existence of diverse populations of mature oligodendrocytes (Tomassy and Fossati, 2014). Interestingly, the authors also discuss recent evidence indicating that neuronal populations modulate the functions of oligodendrocytes, regulating for instance their capacity to produce myelin.

Not only cells from the neural lineage, but also microglial cells emerge as diverse and plastic players in the nervous system. The original article by Xavier et al. reveals a rich heterogeneous microglial population within the SVZ neurogenic niche, raising intriguing possibilities of interactions between microglia and neural progenitor cells (Xavier et al., 2015).

Therefore, this issue covers progenitor diversity in the generation of neurons (embryonic and postnatal), astrocytes and oligodendrocytes, their sites of origin and the spatio-temporal cues regulating their appearance and cell fate determination. The issue also highlights the interplay between intrinsic and extrinsic factors on cell type commitment, opening new perspectives to our understanding of neurodevelopmental disorders, CNS response to lesions, and possible regenerative approaches.

## Conflict of interest statement

The authors declare that the research was conducted in the absence of any commercial or financial relationships that could be construed as a potential conflict of interest.
